# Angiogenesis in the Normal Adrenal Fetal Cortex and Adrenocortical Tumors

**DOI:** 10.3390/cancers13051030

**Published:** 2021-03-01

**Authors:** Sofia S. Pereira, Sofia Oliveira, Mariana P. Monteiro, Duarte Pignatelli

**Affiliations:** 1 Department of Anatomy, Instituto de Ciências Biomédicas Abel Salazar, University of Porto, 4050-313 Porto, Portugal; sspereira@icbas.up.pt (S.S.P.); up201909110@fc.up.pt (S.O.); mpmonteiro@icbas.up.pt (M.P.M.); 2 Clinical and Experimental Endocrinology, Multidisciplinary Unit for Biomedical Research (UMIB), 4050-313 Porto, Portugal; 3 Instituto de Investigação e Inovação em Saúde (I3S), Universidade do Porto, 4200-135 Porto, Portugal; 4 Institute of Molecular Pathology and Immunology, University of Porto (IPATIMUP), 4200-135 Porto, Portugal; 5 Faculty of Sciences, University of Porto, 4169-007 Porto, Portugal; 6 Department of Endocrinology, Hospital S. João, 4200-319 Porto, Portugal; 7 Department of Biomedicine, Faculty of Medicine, University of Porto, 4200-319 Porto, Portugal

**Keywords:** angiogenesis, adrenal fetal cortex, adrenocortical tumors, adrenocortical carcinoma, anti-angiogenic drugs

## Abstract

**Simple Summary:**

Pharmacological angiogenesis modulation was robustly demonstrated to be a powerful clinical resource in oncotherapy. Adrenocortical carcinomas (ACC) often have a poor prognosis for which therapeutic options are limited. Understanding the mechanisms that regulate adrenocortical angiogenesis both under physiological conditions and in ACC could provide important clues on how these processes could be modulated for clinical purposes. This report summarizes the current knowledge on adrenal cortex angiogenesis regulation in physiological conditions and ACC. Embryonic adrenal angiogenesis is regulated by VEGF and Ang-Tie signaling pathways. VEGF angiogenic pathway was initially considered a promising therapeutic target for improving ACC prognosis. However, every single VEGF pathway-targeting clinical trial in ACC so far conducted yielded disappointing results. In contrast, the potential of Ang-Tie pathway-targeting in ACC is yet to be explored. Therefore, further investigation on the role and efficacy of modulating both Ang-Tie and VEGF pathways in ACC is still an unmet need.

**Abstract:**

Angiogenesis plays an important role in several physiological and pathological processes. Pharmacological angiogenesis modulation has been robustly demonstrated to achieve clinical benefits in several cancers. Adrenocortical carcinomas (ACC) are rare tumors that often have a poor prognosis. In addition, therapeutic options for ACC are limited. Understanding the mechanisms that regulate adrenocortical angiogenesis along the embryonic development and in ACC could provide important clues on how these processes could be pharmacologically modulated for ACC treatment. In this report, we performed an integrative review on adrenal cortex angiogenesis regulation in physiological conditions and ACC. During embryonic development, adrenal angiogenesis is regulated by both VEGF and Ang-Tie signaling pathways. In ACC, early research efforts were focused on VEGF signaling and this pathway was identified as a good prognostic factor and thus a promising therapeutic target. However, every clinical trial so far conducted in ACC using VEGF pathway- targeting drugs, alone or in combination, yielded disappointing results. In contrast, although the Ang-Tie pathway has been pointed out as an important regulator of fetal adrenocortical angiogenesis, its role is yet to be explored in ACC. In the future, further research on the role and efficacy of modulating both Ang-Tie and VEGF pathways in ACC is needed.

## 1. Introduction

Angiogenesis is a dynamic process during which new blood vessels are formed derived from pre-existing vasculature. Angiogenesis is an extensively studied process in tumors and a well-recognized hallmark of cancer [1]. Angiogenesis was previously studied in adrenocortical carcinomas (ACC), although the relative rarity of these tumors represents a limitation to conduct extensive clinical and molecular characterization studies. This review aims to bring together all the available data on angiogenesis regulation during the adrenocortical development and in ACC, which could be potentially useful to identify future research avenues to achieve advances in ACC clinical management and disease prognosis. Data source and study selection approach is described in the Supplementary File S1.

## 2. Angiogenesis Regulation

Angiogenesis plays a central role in several physiological (e.g., fetal development and wound healing) and pathological processes (e.g., vascular overgrowth for tumor expansion and metastasis) [2,3,4]. Angiogenesis, either in normal or tumor tissues, usually occurs via one or more of the following mechanisms:

(1) Sprouting angiogenesis, one the most well characterized mechanism leading to angiogenesis, relies on endothelial cells function specification into either tip or stalk cells. Tip cells are derived from the parent vessel, degrade the basement membrane, extend large filopodia which can sense angiogenic factor gradients, such as vascular endothelial growth factor (VEGF), and migrate along the chemotactic paths. In contrast, stalk cells proliferate behind tip cells to form the sprout body, start the process of lumen formation, and connect with neighboring vessels [5,6,7].
(2) Intussusceptive angiogenesis is a process that consists in the splitting of pre-existing vessels into two new vessels. It starts with the formation of transluminal tissue pillars through the invagination of opposing capillary endothelial cells into the vascular lumen, creating a zone of contact. Commonly, intussusceptive and sprouting angiogenesis are complementary mechanisms [5,8].
(3) Recruitment of endothelial progenitor cells and vasculogenesis, a process through which endothelial progenitor cells are recruited in response to several growth factors, cytokines and/or hypoxia-inducible factors. Endothelial progenitor cells differentiate into mature endothelial cells and are incorporated into the angiogenic sprout, thus contributing to new blood vessel formation [4,9].
(4) Vasculogenic mimicry: malignant tumor cells form de novo vessel-like structures without endothelial cells. The newly formed channels mimic the embryonic vascular network pattern, being able to provide enough blood supply to the tumor tissue [10,11].

Multiple signaling pathways regulate blood vessel growth and maintenance. Among these, VEGF and Ang-Tie pathways are particularly important and have been the focus of multiple studies, especially in the context of cancer [12]. VEGF receptor and Tie ligands are widely distributed and were shown to play a coordinated role in endothelial cell proliferation and vessel wall assembly in normal and pathological conditions. 

### 2.1. VEGF Pathway in Angiogenesis Regulation

In mammals, the VEGF system mainly includes five secreted ligands (VEGF-A, VEGF-B, VEGF-C, VEGF-D and placental growth factor) and three primary tyrosine kinase receptors (VEGF-R1, VEGF-R2, VEGF-R3) [13]. The VEGF system also includes the cell-surface proteins, heparan sulfate proteoglycans and neuropilin-1 and -2, which operate as VEGF coreceptors [14,15]. 

VEGFR-1 and VEGFR-2 are expressed in vascular endothelial cells, while VEGF-R3 seems to be prominently expressed in lymphatic endothelial cells [16]. VEGF ligands have different affinities for one of the three VEGF-R. As tyrosine kinases receptors, upon dimerization by a VEFG ligand, the VEGF-Rs auto-phosphorylate, a phenomenon which in turn activates downstream signaling pathways including mitogen-activated protein kinase (MAPK) pathway, the phosphatidylinositol-3 kinase (PI3K-AKT) pathway, and the phospholipase-C-γ pathway. Those pathways drive various intracellular effects in endothelial cells, such as migration, proliferation, and cell survival. The activation of phospholipase-C-γ pathway via VEGF-A-VEGFR-2 binding was reported to be a key signal for endothelial proliferation [17].

In 2001, a new VEGF was identified, the endocrine-gland-derived VEGF (EG-VEGF). This ligand does not show any structural homology to the VEGF family, but displays several biological similarities to VEGF ligands, including hypoxic regulation and ability to induce fenestration in target cells. Moreover, EG-VEGF expression is restricted to steroidogenic tissues (adrenal, ovary, testis and placenta) and its effects seem to be restricted to endothelial cells derived from these organs [18].

### 2.2. Ang-Tie Pathway in Angiogenesis Regulation

**Figure 1:**
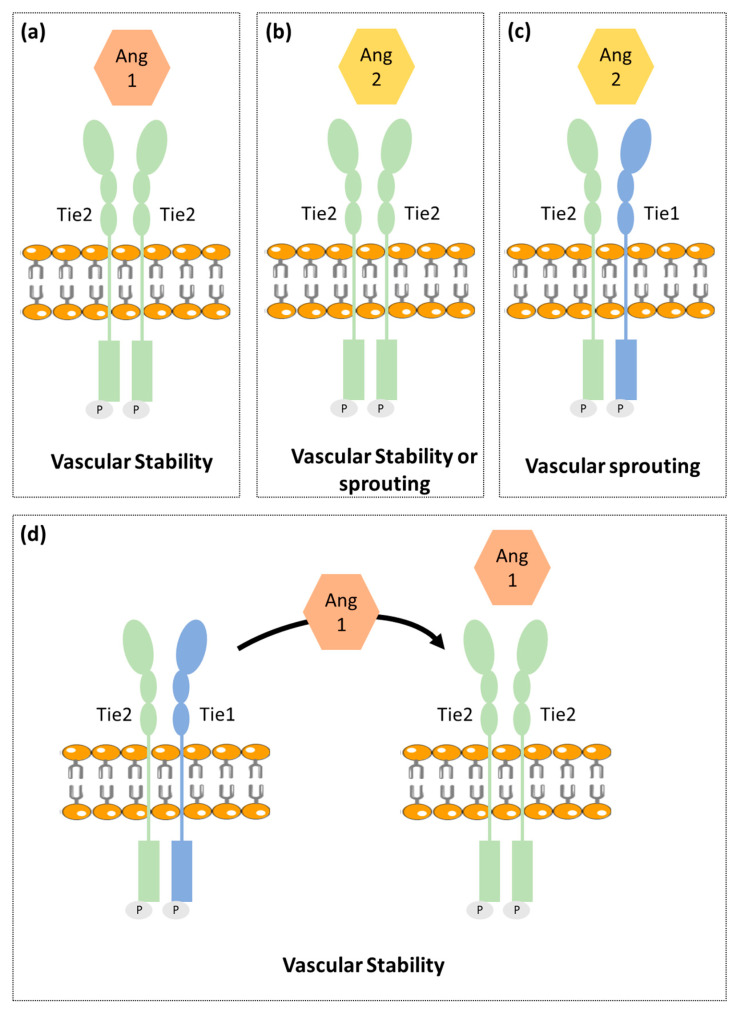
Schematic illustration of the vessel stability regulation by Ang/Tie signaling. (**a**) Ang1 binds to Tie2 promoting vascular stability. (**b**) Ang2 has a dual function acting as a Tie2 agonist or antagonist to promote vascular stability or sprouting, respectively. (**c**) Tie1 forms a complex with Tie2. Upon Ang2 stimulation, Tie1 and Tie2 remain associated and Ang2 induces vascular sprouting. (**d**) Ang1 stimulation promotes Tie2 clustering leading to vascular stability. This schematic representation includes the most consensual theories; however, this pathway is not yet fully understood. Besides that, this figure is a schematic representation that is not intended to translate the real chemical conformation of the proteins.

Ang-Tie signaling pathway regulates vascular permeability and remodeling during tumor angiogenesis and metastasis. Ang/Tie signaling seems to complement the VEGF signaling pathway by controlling later stages of angiogenesis and by being involved in vascular maturation (Figure 1) [19].

The angiopoietin family includes two type 1 transmembrane protein receptors: Tie1 and Tie2 and four ligands: Ang1, Ang2, Ang3 and Ang4. Ang1 and Ang2 have been identified as the main ligands for Tie receptors, while the Ang3 and Ang4 biological function is still poorly characterized [20,21,22].

Ang1 binds and activates Tie2 resulting in Tie2 internalization and ligand release. Then it leads to Tie2 tyrosine residues phosphorylation that in turn recruits adaptor proteins and ignites PI3K/Akt and MAPK signaling pathways, promoting pro-survival, anti-permeability, and anti-inflammatory effects on endothelial cells [23]. Tie2 is not required for the endothelial cells’ differentiation but is rather reported as necessary for cell maintenance [24].

Ang2 that shares approx. 60% amino acid homology with Ang1, binds to Tie2 with a similar affinity as Ang1. Ang2 seems to block the Ang1-induced Tie2 phosphorylation. Ang2 is upregulated during tumor angiogenesis and so was considered as a potential antiangiogenic target. However, recent studies found Ang2 to have a dual function, acting as a Tie2 antagonist in the presence of Ang1 or acting as a Tie2 agonist in the absence of Ang1 [25]. Different studies reported that it is unlikely that Tie2 can act differently when binding to Ang1 and Ang2, since both angiopoietins interact with Tie2 in a structurally similar manner and pointed out that other still unidentified mechanisms were likely to be involved [26]. One of the proposed mechanisms involve the Tie1 receptor [27]. 

Contrary to Tie2, the Tie1 has been less well characterized. Tie1 is considered an orphan receptor and is mainly expressed at vascular bifurcations and branching points, with no yet identified in vivo ligand [28]. It is well known, however, that Tie1 has an important role in vascular development, since its inactivation causes late embryonic lethality and vasculature maturation failure [29,30]. Recent studies proposed that Tie1 forms a complex with Tie2 on the endothelial cell surface and acts as a Tie2 inhibitor [27]. Cells expressing both receptors are responsive to chemotactic signals and able to promote vessel branching and sprouting that is required for angiogenesis. On the other hand, Tie1 is absent in stable and quiescent mature vessels [27]. 

A mechanistic study indicated Tie1 as being responsible for angiopoientin’s differential function. In mature vessels, as Tie1 is absent, Tie2 can be activated by either Ang1 or Ang2, to promote vessel stability. On active angiogenesis sites, Tie1 and Tie2 form a complex and Ang2 fails to activate Tie2, allowing vessel branching to be promoted. On the other hand, Ang1 is able to dissociate Tie2 from the Tie1-Tie2 complex, activating Tie2 and thus enhancing vascular stability [27,31].

## 3. Angiogenesis in Normal Adrenal Cortex

### 3.1. Fetal Adrenal Cortex

Human fetal adrenal (HFA) plays a critical role in fetal maturation and perinatal survival. HFA steroid hormones regulate intrauterine homeostasis and appropriate fetal organ systems maturation [32,33].

Contrary to the adult adrenal cortex that includes three distinct zones: glomerulosa, fasciculata and reticularis; the HFA is primarily composed of two single distinct zones: outer zone or definitive zone and inner zone or fetal zone [33,34]. The definitive zone comprises a narrow band of small cells that exhibit typical characteristics of cells in proliferative state. Definitive zone does not produce steroids until the third trimester. However, as gestation advances, definitive zone cells start to accumulate lipids and resemble steroidogenic active cells. The fetal zone is the largest adrenal cortex zone and consists of large cells that exhibit features characteristic of steroid-secreting cells [33,34,35,36,37]. In ultrastructural studies, a third zone in between definitive zone and fetal zone, named transitional zone, has been described. The transitional zone is composed by cells with intermediate characteristics, but capable to synthetize cortisol and so cells can be considered analogous to fasciculata layer cells of mature adrenal cortex [33,38,39,40,41]. 

Due to the HFA critical role in fetal maturation, the early and extensive vasculature development that occurs in this gland, is not only necessary but also particularly important. Angiogenesis is not only required for HFA growth and maturation, but it is also necessary for the influx of steroid precursors and trophic factors into the gland to enable mature steroids synthesis and secretion into circulation. Indeed, the fetal adrenal gland is one of the most highly vascularized organs in the human fetus [41]. 

Previous studies have reported that VEGF-A, FGF-2, Ang1, Ang2, and Tie2 are expressed in HFA since midgestation and to have a putative role in adrenal gland angiogenesis [34,42,43]. 

Ang2 expression in HFA is markedly higher when compared to the mature adrenal gland, whereas Ang1 and Tie2 expression seem to be similar in both fetal and adult adrenals. Thus, supporting higher angiogenesis activity and vascular instability in developing adrenal glands [42]. 

Ang2, FGF-2 and VEGF-A expression are mainly expressed in the gland periphery suggesting that the HFA periphery is the primary site of angiogenesis, in parallel to cell proliferation [42,43]. Further supporting this hypothesis, a dense network of irregular capillaries was also observed at the HFA periphery [44].

On the contrary, Ang1 is mainly expressed in the fetal zone, suggesting that the inner adrenal zone presents a greater vessel maturity. Tie2, was exclusively identified to be present in endothelial cells throughout the gland [42,43]. 

Adrenocorticotropic hormone (ACTH), the main regulator of HFA growth and function, also seems to be implicated in angiogenesis control. In vitro studies found that ACTH upregulates VEGF-A, FGF-2 and Ang2 in the HFA, therefore controlling angiogenesis while simultaneously exerting growth and secretion stimulatory actions [42,43,45,46]. 

The steroidogenic factor 1 has a critical role in adrenal development, steroidogenesis, and also in gonadal differentiation [47]. In addition, steroidogenic factor 1 also seems to be implicated in HFA angiogenesis regulation by direct interaction and activation of the Ang2 gene promoter. Furthermore, the authors demonstrated that steroidogenic factor 1 and Ang2 are strongly co-expressed in HFA periphery in early stages of development [48].

Overall, these findings support that the adrenal gland growth, steroidogenesis and blood vessel formation, are synchronized phenomena [42,43,45,46].

### 3.2. Adult Adrenal Cortex

The adrenal gland is one of the most vascularized organs in adult mammalian organisms. Its developed intrinsic vasculature is required for an efficient secretion of steroid hormones into the systemic blood flow. The adrenal gland is supplied by three different arterial branches derived the abdominal aorta: inferior phrenic artery, middle adrenal artery and renal artery. The arterial blood enters in the adrenal gland and flows centripetally through the adrenal cortex into the adrenal medulla [49,50].

Previous studies have found that adrenocortical cells highly express VEGF-A and EG-VEGF—a VEGF specific of steroidogenic organs, both having been pointed out as important molecules for maintenance of the dense and fenestrated vasculature of the adrenal cortex. This expression also seems to be regulated by ACTH [51,52,53,54,55]. 

In addition, the vasculature of the adrenal cortex seems to be coordinated with the mass of the adrenal cortex, since it suffers fluctuations decreasing or increasing along regression or expansion of the adrenal cortex, respectively [52]. 

## 4. Angiogenesis in Adrenocortical Tumors

Adrenocortical tumors (ACT) are common adrenal tumors affecting 3% to 10% of the human population [56]. The majority of ACT are benign non-functioning adrenocortical adenomas (ACA), while malignant ACC are rare with an incidence of 0.7 to 2 per million per year [56]. ACC most often have a poor prognosis and are frequently already metastasized when first diagnosed. ACC pathogenesis is still largely unclear, which results in a lack of biomarkers available for diagnosis and in limited treatment options [57,58]. 

The status of the VEGF pathway in adrenocortical tumors has been already addressed in multiple studies (Table 1).

Patients with ACT were found to present higher VEGF serum levels as compared to healthy controls [59,60]. In addition, Kolomecki et al. demonstrated that VEGF serum levels were significantly higher in patients with non-functioning malignant tumors than in patients with non-functioning ACA. Noteworthy, VEGF serum levels in patients with ACC were shown to decrease after tumor surgical resection and increase in patients who experienced tumor recurrence [59]. de Fraipont et al. found that cytosolic VEGF-A concentrations were higher in ACC when compared to ACA, although not being significantly different when localized and more invasive ACC were compared [63]. Nevertheless, cytosolic VEGF-A concentrations were higher in recurrent as compared to non-recurrent ACC after primary tumor resection [63].

Tumor VEGF expression was also found to be higher in ACC as compared to normal adrenal glands and ACA [61,62,64]. VEGF receptor 2 tumor expression was also found to be higher in ACC when compared with ACA and normal adrenal glands [62,64].

Bernini et al., however, found that tumor VEGF expression was not directly related with vascular density, which was lower in ACC as compared to ACA and normal adrenal tissue. The fact that a higher VEGF expression was not shown to be associated with increased vascular density in ACC, was somehow unsurprising since a high vascular density already characterizes normal adrenal cortex tissue. What surprised researchers was that despite ACC lower vascular density, patients still had a very short survival time [61].

Other studies reported that although no differences in vascular density were noticed when ACC, ACA and normal adrenal glands were compared, blood vessels perimeter and area were higher in ACC when compared to ACA [65,66]. In addition, endothelial cell proliferation was higher in ACC [66]. 

On an opposed direction, another group reported vascular density to be higher in malignant ACT as compared to benign ACT [67]. Another study observed that in their series VEGF expression was positively correlated with vessel density [64]. Pereira et al. also reported ACC to present a higher vascular density, but only when compared to cortisol secreting ACA [68]. This could, however, be derived from cortisol anti-angiogenic effects [69]. There is additional evidence supporting that adrenocortical angiogenic status could be tightly related to the tumor’s hormonal functionality. Bernini et al. found that VEGF tumor expression was higher in aldosterone secreting ACA as compared to non-functioning ACA and normal adrenal glands [61]. In addition, in another study patients with cortisol-secreting ACA were found to have higher circulating VEGF levels than patients with aldosterone secreting adenomas [60]. 

The discovery of EG-VEGF, a steroidogenic organ specific VEGF, brought some enthusiasm to the scientific community as a potential explanation to the contradictory angiogenic patterns in ACTs as well as a potential target for ACC treatment. Heck et al. characterized the expression of EG-VEGF and its receptors [prokineticin receptor 1 (PKR1) and 2 (PKR2)] in a large number of ACC, ACA and normal adrenal glands. In this study, EG-VEGF and both receptors PKR1 and PKR2 were found to be present in the majority of ACT. Moreover, the nuclear protein expression of either EG-VEGF or PKR1 or both in ACC was reported to be associated with higher mortality, suggesting that these could be used as prognostic markers for overall patient survival [53]. 

New prognostic and diagnostic markers are needed to improve ACC clinical practice. As described in this section, the usefulness of angiogenic factors for ACC diagnosis and/or prognosis was already investigated. From those, VEGF was the one with more consistent and replicable results, being increased in ACC when compared with ACA [59,61,63,64], in particular in the recurrent malignant tumors [60,63]. However, due to the rarity of ACC, the number of patients included in each study is small. So, in the future, to validate this result, multi-center studies are needed to increase the samples/participants’ number and to uniformize the methodological approach to analyze the VEGF tumors expression in ACT. Stratified analysis according to tumors functionality are needed since in previous studies, it showed to influence VEGF levels. 

## 5. Anti-Angiogenic Agents’ Efficacy in Adrenocortical Carcinomas Treatment

The demonstration that patients with ACC had high VEGF circulating levels and tumor expression, along with the recent evidence on the efficacy of anti-VEGF drugs for other types of neoplasia treatment, such as, advanced colorectal cancer [70], opened the promising perspective of using this drug class agents for ACC treatment as well.

The first report using an anti-angiogenic agent for the treatment of patients with ACC was released in 2010 (Table 2). Ten patients with advanced ACC, refractory to several cytotoxic chemotherapies, were treated with the monoclonal anti-VEGF antibody bevacizumab in combination with capecitabine, an adrenolytic agent. The results were disappointing since the disease progressed in all patients [71].

A phase II clinical trial using sorafenib in combination with paclitaxel was conducted in ten patients with advanced ACC after treatment with mitotane plus one or two chemotherapy lines. Sorafenib is a tyrosine kinase inhibitor (TKI) drug that inhibits several receptors, such as VEGFR2, VEGFR3, platelet-derived growth factor receptor (PDFGR) and RAF-1, a key enzyme in the MAPK-ERK signaling pathways. The sorafenib plus paclitaxel drug combination was demonstrated to be ineffective in patients with ACC, as progressive disease was observed in nine consecutive patients leading to clinical trial interruption 2 months after initiation [72].

In another trial 35 patients with ACC refractory to mitotane and cytotoxic chemotherapies were treated with the TKI sunitinib, a drug that inhibits multiple receptors, such as VEGFR1 and VEGFR2, c-KIT, Fms-like tyrosine kinase 3, and PDGFR. Six of the thirty-five patients in that trial died of progressive disease. Of the remaining twenty-nine patients, five patients had stable disease, and 23 patients had progressive disease on first evaluation (12 weeks). Three of the five patients with stable disease on first evaluation, had disease progression later. In addition, authors reported that concomitant mitotane had a negative impact on treatment outcome, by lowering sunitinib blood levels. Therefore, sunitinib only demonstrated to have a modest efficacy in the treatment of patients with advanced ACC, while the efficacy in patients without mitotane exposure needs to be further assessed [73]. 

In a phase II clinical trial, axitinib, a potent VEGFR-1, VEGFR-2, and VEGFR-3 selective inhibitor, was administrated to thirteen patients with metastatic ACC previously treated with at least one chemotherapy regimen, with or without mitotane. No patient in trial achieved a partial or complete response, and only eight patients experienced stable disease for more than 3 months [74]. 

Thalidomide is an immunomodulatory agent with anti-angiogenic properties by targeting TNF-α, ILs, VEGF, bFGF. The effectiveness of thalidomide was investigated in a trial that included twenty-seven patients with advanced ACC refractory to mitotane and other systemic drug treatments. Twenty-five of the twenty-seven patients experienced clinical or radiological disease progression at the time of first evaluation. So, thalidomide also, only showed to be marginally effective in patients with refractory advanced ACC [75].

Lenvatinib is another TKI drug that inhibits multiple receptors including VEGFR-1, VEGFR-2 and VEGFR-3, FGFRs, PDGFR-α, KIT and RET. The efficacy of lenvatinib in combination with pembrolizumab, an immune checkpoint inhibitor, was also investigated in eight patients with ACC and progressive and/or metastatic disease after receiving previous treatment interventions. None of the eight patients had to discontinue the treatment in result of toxicity. One patient had stable disease, lasting for 8 months, two patients had a partial response while receiving therapy and five patients developed progressive disease, so lenvatinib plus pembrolizumab combined therapy was demonstrated to achieve positive responses in a subset of patients without significant toxicity [76]. Phase II clinical trials with larger patient cohorts are still needed to confirm these conclusions. 

The clinical efficacy and safety of the TKI cabozantinib was investigated in a retrospective cohort study in sixteen patients with advanced ACC after other treatments having failed. Cabozantinib is a multi-inhibitor of c-MET, VEGFR-2, AXL, and RET. At first evaluation, two patients had partial response and six had stable disease. At four months evaluation, half of patients were alive and progression free [77]. Although these results were not brilliant, they were superior to the ones previously reported for other anti-angiogenic agents. 

Although previous studies using anti-angiogenic therapies did not show encouraging results in patients with ACC, there are several registered clinical trials using VEGF-R inhibitors ongoing or due to be initiated. Two phase II clinical trials designed to test the efficacy of cabozantinib in patients with advanced ACC (NCT03370718 and NCT03612232) are currently recruiting, and a phase II clinical trial to test the efficacy of the VEGFR-2 inhibitor apatinib plus camrelizumab an immune checkpoint inhibitor, is registered (NCT04318730). 

**Table 2:** Clinical studies using anti-angiogenic drugs for the treatment of patients with adrenocortical carcinomas.

Anti-Angiogenic Drug	Mechanism of Action	Study Type	Patient Population	Results	Ref.
Bevacizumab (+capecitabine)	Monoclonal anti-VEGF antibody	Observational retrospective cohort study	Patients with refractory ACC (*n* = 10)	PFS: 59 days OS: 124 days	[71]
Thalidomide	Immunomodulatory agent that targets TNF-α, ILs, VEGF, bFGF	Observational retrospective cohort study	Patients with refractory ACC (*n* = 27)	PFS: 11.2 weeks (4.4–22.8 weeks) OS: 36.4 weeks (5.1–111.1 weeks)	[75]
Lenvatinib (+pembrolizumab)	Multi-TKI that inhibits VEGFR-1, VEGFR-2 and VEGFR-3, FGFRs, PDGFR-α, KIT, RET	Observational retrospective cohort study	Patients with recurrent and/or metastatic ACC (*n* = 8)	PFS: 5.5 months OS: NA	[76]
Cabozatinib	TKI that targets VEGFR-2 and c-Met	Observational retrospective cohort study	Patients with refractory metastatic ACC (*n* = 16)	PFS: 16.2 weeks (2.8–61 weeks) OS: 56 weeks (5.6–83.1 weeks)	[77]
Sorafenib (+paclitaxel)	Multi-TKI inhibitor that VEGFR-2 VEGFR-3, PDGFR and RAF-1	Phase II, single-arm, open label clinical trial	Patients with refractory metastatic ACC (*n* = 10)	Trial interrupted due disease progression in all enrolled patients	[72]
Sunitinib	Multi-TKI that inhibits VEGFR-1 and VEGFR-2, c-KIT, FLT3 and PDGFR	Phase II, single-arm, open label clinical trial	Patients with advanced ACC after mitotane or others cytotoxic drugs (*n* = 35)	PFS: 2.8 months (5.6–11.2 months) OS: 5.4 months (14.0–35.5 months)	[73]
Axitinib	Selective inhibitor of VEGFR-1, VEGFR-2 and VEGFR-3	Phase II, single-arm, open label clinical trial	Patients with metastatic ACC previously treated with at least one chemotherapy regimen (*n* = 13)	PFS: 5.48 months (1.8–10.92 months) OS: 13.7 months	[74]

FPS and OS median and ranges were included in the table, when available in the original manuscript. ACC—Adrenocortical carcinoma; bFGF—basic fibroblast growth factor; FGFRs—fibroblast growth factor receptors; FLT3—FMS-like tyrosine kinase 3; Ils—interleukins; NA—not available; OS—Overall Survival; PFS—Progression-free survival; TKI—Tyrosine kinase inhibitor; TNF-α—Tumor necrosis factor alpha; VEGF—Vascular endothelial growth factor; VEGFR—Vascular endothelial growth factor receptor; PDGFR—platelet-derived growth factor receptor.

It is important to highlight that although the VEGF pathway is one of the most important angiogenic pathways, this is not the only pathway involved in angiogenesis regulation. As far as we are aware, no clinical trial has tested the effect of drugs targeting the Ang-Tie pathway in patients with ACC.

In 2014, one single patient with ACC was enrolled in a phase 1b clinical trial designed to test the efficacy of trebananib, a dual Ang1 and Ang2 inhibitor plus VEGFR inhibitors (bevacizumab or motesanib) in various solid tumors. Stable disease was achieved in the patient with ACC. However, since this clinical trial included patients with different solid tumors no further data specifically related to this patient is available [78].

Further investigation of molecules involved in Ang-Tie pathway in ACC tissues is needed in order to understand whether this pathway has a role in the pathophysiology of this type of tumor. This knowledge is needed to provide a rationale for conducting clinical trials targeting both Ang-Tie and VEGF pathways in patients with ACC.

There are no doubts that angiogenesis is an important process in ACC progression and the rationale for the use of anti-angiogenic drugs in ACC treatment is unquestionable. However, so far clinical trials to test the efficacy of these drugs were conducted in patients with advanced/metastatic ACC that precluded the possibilities of success, since even if the angiogenic capacity of the tumor is decreased, the disease is already in an uncontrolled stage. In contrast, the efficacy of anti-angiogenic drugs in non-advanced ACC is unknown yet. Therefore, ex-vivo studies could be useful to assess these drugs efficacy as compared to mitotane, which is the only drug licensed for ACC treatment.

In addition, whenever possible, conducting studies to evaluate whether there is a correlation between drug efficacy and the tumor molecular profile, would provide additional insights on the mechanisms responsible for successful or unsuccessful treatment and support future clinical trials.

## 6. Conclusions

Angiogenesis is well-known to be required for cancer cell expansion and considered an important hallmark of cancer [1]. Adrenal glands have a very dense vascular network that is necessary to support their hormonal secretion functions. Therefore, it may not be surprising that this normal adrenal gland high vascular density is unlikely to be further increased in the context of adrenocortical neoplasia.

Although several molecules within the VEGF pathway were identified as prognostic markers and promising targets for ACC treatment, the cohort studies and clinical trials so far concluded yielded disappointing results. The most promising results were observed for cabozantinib, a multi-TKI inhibitor, which induced stable or partial response in half of the patients with advanced ACC [77]. Given this effect and its overall safety with a tolerable side effect profile, two clinical trials to assess the efficacy of this drug are now recruiting (NCT03370718 and NCT03612232). Both trials require the discontinuation of mitotane and exclude patients with mitotane levels higher than 2 mg/L. Future clinical trials should take in consideration prior mitotane exposure on the study design, since previous or concomitant mitotane use may influence the investigational drug treatment outcomes due to its impact on drug metabolism [73,79].

Another registered clinical trial will assess the efficacy of the anti-angiogenic drug Apatinib combined with an immunomodulatory agent (PD-1 inhibitor: camrelizumab) in ACC (NCT04318730), a drug that was previously tested alone and was demonstrated to induce a stable disease or an objective response in 52% of the patients [80]. Since this drug combination elicited impressive clinical results in many solid tumors [81,82,83], there is a great expectation for this clinical trial outcomes.

Furthermore, the Ang-Tie pathway which is known to have an important role on fetal adrenal gland angiogenesis should also receive attention [42,43]. The role of Ang-Tie pathway in adrenocortical tumors has not yet been investigated. Future studies and clinical trials investigating the role of Ang-Tie pathway in adrenocortical tumors and the efficacy of targeting Ang-Tie or both Ang-Tie and VEGF pathways in ACC treatment are needed.

## Figures and Tables

**Table 1:** VEGF pathway findings in adrenocortical tumors.

	Patient Group Comparisons	Results
VEGF	Patients with ACT vs. Healthy individuals	↑ VEGF serum levels in patients with ACT [59,60]
	Aldosterone secreting ACA vs. Non-functioning ACA	↑ VEGF tumor expression in aldosterone producing ACA [61]
	Cortisol secreting ACA vs. Aldosterone secreting ACA	↑ VEGF serum levels patients with cortisol secreting ACA [60]
	ACC vs. Normal adrenal glands	↑ VEGF expression in ACC [61,62]
	ACC vs. ACA	↑ VEGF serum levels in ACC ↑ VEGF tumor expression in ACC [59,61,63,64]
	Patients with recurrent ACC vs. Patients with non-recurrent ACC	↑ VEGF serum levels in recurrent ACC ↑ VEGF tumor expression in recurrent ACC [60,63]
	Localized ACC vs. Invasive ACC	No difference in VEGF tumor expression [63]
VEGF-R2	ACC vs. Normal adrenal glands	↑ VEGF-R2 tumor expression in ACC [62]
	ACC vs. ACA	↑ VEGF-R2 tumor expression in ACC [64]

ACA—Adrenocortical Adenomas; ACC—Adrenocortical carcinomas; ACT—Adrenocortical tumors; VEGF—Vascular endothelial growth factor; VEGFR—Vascular endothelial growth factor receptor; ↑—Increased protein levels or expression.

## Supplementary Materials

The following are available online at https://www.mdpi.com/2072-6694/13/5/1030/s1. Supplementary File S1: Data Source and Study Selection.
