# Supplementary material for: Angiogenesis in the Normal Adrenal Fetal Cortex and Adrenocortical Tumors
Source: Cancers (Basel). 2021 Mar 1;13(5):1030. doi: 10.3390/cancers13051030 (PMC7957756; doi:10.3390/cancers13051030)
Supplement: Supplementary file 1 [file cancers-13-01030-s001.pdf]

Supplementary file 1: Data Source and Study Selection.

To review the current knowledge on adrenal cortex angiogenesis regulation in physiological conditions and in adrenocortical carcinoma, we conducted a PubMed search of all English-language papers that were published in MEDLINE, until December 2020.

The following search terms were used, combined with Boolean operators: adrenal gland OR adrenal cortex OR fetal adrenal OR adrenal tumor OR adrenocortical tumor OR adrenocortical cancer OR adrenocortical carcinoma OR adrenocortical adenoma AND angiogenesis OR angiogenic OR vascularization OR vasculature OR vascular endothelial growth factor OR VEGF OR ANG OR Tie. Manuscripts were excluded if: not performed in adrenocortical tumors; not related to angiogenesis; not performed in humans or performed in cell lines. Since adrenocortical carcinomas are rare tumors, the number of samples/participants in each study was not used as a reason to exclude a manuscript.

An additional search was performed in ClinicalTrials.gov to find the clinical trials registered for adrenocortical carcinoma using anti-angiogenic therapies.
